# Identification of High Molecular Variation Loci in Complete Chloroplast Genomes of *Mammillaria* (Cactaceae, Caryophyllales)

**DOI:** 10.3390/genes11070830

**Published:** 2020-07-21

**Authors:** Delil A. Chincoya, Alejandro Sanchez-Flores, Karel Estrada, Clara E. Díaz-Velásquez, Antonio González-Rodríguez, Felipe Vaca-Paniagua, Patricia Dávila, Salvador Arias, Sofía Solórzano

**Affiliations:** 1 Laboratorio de Ecología Molecular y Evolución, Unidad de Biotecnología y Prototipos FES Iztacala, Universidad Nacional Autónoma de México, Avenida de los Barrios 1, Los Reyes Iztacala, Tlalnepantla de Baz 54090, Mexico; dela@comunidad.unam.mx; 2 Instituto de Biotecnología, Unidad Universitaria de Secuenciación Masiva y Bioinformática, Universidad Nacional Autónoma de México, Avenida Universidad 2001, Chamilpa, Cuernavaca 62250, Mexico; alexsf@ibt.unam.mx (A.S.-F.); karel@ibt.unam.mx (K.E.); 3 Laboratorio Nacional en Salud, Diagnóstico Molecular y Efecto Ambiental en Enfermedades Crónico-Degenerativas, FES Iztacala, Universidad Nacional Autónoma de México, Los Reyes Iztacala, Tlalnepantla de Baz 54090, Mexico; cdiaz@comunidad.unam.mx (C.E.D.-V.); felipe.vaca@iztacala.unam.mx (F.V.-P.); 4 Laboratorio de Genética de la Conservación, Instituto de Investigaciones en Ecosistemas y Sustentabilidad, Universidad Nacional Autónoma de México, Antigua Carretera a Pátzcuaro 8701, Ex-Hacienda San José La Huerta, Morelia 58190, Mexico; agrodrig@cieco.unam.mx; 5 Subdirección de Investigación Básica, Instituto Nacional de Cancerología, Ciudad de México 04510, Mexico; 6 Laboratorio de Recursos Naturales, Unidad de Biotecnología y Prototipos, FES Iztacala, Universidad Nacional Autónoma de México, Avenida de los Barrios 1, Los Reyes Iztacala, Tlalnepantla de Baz 54090, Mexico; pdavilaa@unam.mx; 7 Jardín Botánico, Instituto de Biología, Universidad Nacional Autónoma de México, Tercer Circuito Exterior, Ciudad Universitaria, Coyoacán, Ciudad de México 04510, Mexico; sarias@ib.unam.mx

**Keywords:** locus, *Mammillaria*, molecular variation, non-coding regions, repeated sequences, SSRs

## Abstract

In plants, partial DNA sequences of chloroplasts have been widely used in evolutionary studies. However, the Cactaceae family (1500–1800 species) lacks molecular markers that allow a phylogenetic resolution between species and genera. In order to identify sequences with high variation levels, we compared previously reported complete chloroplast genomes of seven species of *Mammillaria*. We identified repeated sequences (RSs) and two types of DNA variation: short sequence repeats (SSRs) and divergent homologous loci. The species with the highest number of RSs was *M. solisioides* (256), whereas *M. pectinifera* contained the highest amount of SSRs (84). In contrast, *M. zephyranthoides* contained the lowest number (35) of both RSs and SSRs. In addition, five of the SSRs were found in the seven species, but only three of them showed variation. A total of 180 homologous loci were identified among the seven species. Out of these, 20 loci showed a molecular variation of 5% to 31%, and 12 had a length within the range of 150 to 1000 bp. We conclude that the high levels of variation at the reported loci represent valuable knowledge that may help to resolve phylogenetic relationships and that may potentially be convenient as molecular markers for population genetics and phylogeographic studies.

## 1. Introduction

The analysis of DNA variation is critical when studying biological patterns in evolutionary biology, conservation and molecular ecology. For example, the comparison of complete sequences of chloroplast DNA (cpDNA) has enabled the interpretation of its evolutionary history [1] by tracking variations in genes related to the contemporary cyanobacteria. Genes are not randomly arranged in the circular molecule of cpDNA contained in each chloroplast. On the contrary, they are often located in a relatively conserved position that can be associated to each taxonomic group. Moreover, the total number of genes and the total size of cpDNA tend to be evolutionarily conserved, as does the absence or presence of two blocks of genes organized as inverted repeats [2]. Therefore, the information used for evolutionary studies varies depending on the type of plant.

In land flowering plants, the cpDNA is divided into four sections: two single copies (SCs), one of them larger (LSC) than the other one (SSC), which are separated by the two inverted repeats (IRs) [2]. The two IRs are the main source of structural variation within lineages and among the members of large taxonomic groups. The evolutionary information reflected in the structure of cpDNAs has been described [3], and it has been used as characters in phylogenetic studies [4,5]. The other source of variation in cpDNA is at the nucleotide level, comprising substitutions (transitions/transversions), gains or losses of a single or a block of nucleotides (insertions or deletions), and changes in the orientation of a pair or a large block of nucleotides (inverted sequences) [6,7].

In the cpDNA of land plants, there are two types of non-coding sequences, the intergenic spacers (IGSs) and the introns, which are currently most commonly used as molecular markers in phylogenetic studies [8]. In angiosperms, the IGSs *trnK-matK, psbA-trnH, trnL-trnF* [9,10,11] and intron rpl16 [12] are recognized by their high molecular variation. However, across different taxonomic groups of angiosperms these DNA sequences exhibit unequal levels of molecular variation, and in some groups these loci frequently do not show high levels of variation. Consequently, under these conditions these markers are not useful in resolving phylogenetic relationships among species of a single genus or those of closely related genera [13,14,15,16,17]. However, such molecular markers resolved ancient deep divergent processes in distant phylogenetic taxa [18,19].

Thus, it is evident that the levels of molecular variation in non-coding and coding sequences of cpDNA that have for a long time been traditionally used to establish phylogenetic relationships are insufficient for all the taxonomic groups of angiosperms. Hence, it is necessary to find other sources of variation in the nuclear, chloroplast and mitochondrial genomes, in order to resolve phylogenies and other evolutionary issues. In this study, we focused on studying complete chloroplast genomes, since the comparative genomics perspective allows for a better identification of deep structural changes and punctual mutation changes that may provide insight into evolutionary processes. Recently, a high structural variation of chloroplast genomes was identified among species of the short-globose cacti of the genus *Mammillaria* [20], which showed strong differences with respect to the columnar cactus *Carnegiea gigantea* [21]. However, the amount of molecular variation in DNA sequences has not been evaluated in the cpDNA of the rich genus *Mammillaria*. This genus has received attention in previously published phylogenetic studies, but the phylogenetic relationship between the existent species has only been partially resolved, as it also occurs with other related genera. These studies obtained large polytomies, and even species of different genera were placed at the same internal nodes (e.g., [22,23]). Such unresolved phylogeny topologies are a common result for many angiosperm families and are not exclusive to Cactaceae. This is a consequence of a lack of molecular markers in many plant groups exhibiting high levels of variation [13,17].

*Mammillaria* Haw. (Cactaceae, Cactoideae, Cacteae) is the richest cactus genus; it groups 163 species [24]. Besides the diversity relevance, 192 species and subspecies of this genus are a world conservation concern [25]. Previous phylogenetic studies based on *trnL-trnF* [13], *trnK-matK* [22] and *psbA-trnH* and *rpl16* intron [23] did not resolve the phylogenetic relationship within the group. In addition, the study based on the IGS *psbA-trnH* and *rpl16* intron concluded that the members of *Mammillaria* do not have a monophyletic origin [23]. Moreover, these studies did not resolve the phylogenetic relationships of *Mammillaria* to six other cacti genera (*Coryphantha, Cumarina, Escobaria, Neolloydia, Ortegocactus* and *Pelecyphora*). It seems that the low levels of variation found in the molecular markers that were used may be associated to the relatively recent origin of *Mammillaria,* which is estimated to be <8 Ma [26,27]. In addition, the small number of molecular markers used in most of the phylogenetic studies may be an additional factor in explaining the unresolved phylogenies that were obtained. Considering the current scenario, finding molecular markers with the appropriate molecular variation levels in order to resolve the phylogenetic relationship between species and genera remains a challenge. Based on the genomic comparison of the cpDNA of phylogenetically close species of *Mammillaria*, we aimed to identify sources of high molecular variation, assuming that they might fully resolve phylogenetic relationships not only in *Mammillaria* but also in other Cactaceae and Caryophyllales members.

## 2. Materials and Methods 

### 2.1. Data Source

We used the complete chloroplast genome of seven species of *Mammillaria* recently assembled: *M. albiflora, M. crucigera, M. huitzilopochtli, M. pectinifera, M. solisioides, M. supertexta* and *M. zephyranthoides* [20]. The seven chloroplast genomes are uploaded to the NCBI digital database and freely available in genbank (Table S1). By comparing the complete chloroplast genomes of these seven *Mammillaria* species, we identified three types of cpDNA sequences as sources of molecular variation: repeated sequences (RSs), short sequences of the microsatellite type (SSRs) and divergent DNA sequences in homologous loci.

### 2.2. Repeats Characterization and Short Sequence Repeats Identification

We compared the cpDNA of the seven studied species (Table S1) and identified forward, reverse, palindromic and complementary RSs with a minimum size of 30 bp, using a Hamming distance of three with REPUTER [28]. In addition, for each species, SSRs were identified with the MISA perl script [29]. The minimum number of repeat units was ten for homopolymer repeats, six for dinucleotide and five for trinucleotide, tetranucleotide, pentanucleotide and hexanucleotide repeats. 

### 2.3. Sequence Divergence in Homologous Loci Among Species 

A total of 180 homologous DNA regions were identified; of these, 77 were coding and 103 were non-coding sequences. Each of these 180 loci was aligned with MAFFT v7.310 [30] by paired comparisons between species. For each of the aligned loci, the proportion of nucleotide substitutions was documented (total number of divergent changes/locus length). The changes in the nucleotide position considered transitions and transversions. Each gap ≥1 of contiguous bases was counted as a single evolutionary change. The percentage of variation by locus among 21 paired comparisons was obtained and averaged. The percentage of variation by locus was calculated with the formula: % variation = [(NS + ID)/L) × 100], where NS = nucleotide substitutions, ID = number of indels (indels of any size were quantified as 1) and L = locus length.

### 2.4. Phylogenetic Analysis

A total of 14 concatenated loci were used in order to reconstruct the phylogenetic relationships of the seven species. The 14 loci were selected from the 20 most variable homologous loci among the *Mammillaria* species that were also found in the outgroup genomes. The giant columnar cactus *Carnegiea gigantea* (Cactaceae) [21] and purslane *Portulaca oleracea* (Portulacaceae) [31] of the order Caryophyllales were used as outgroups. The sequences of the 14 loci were aligned with MAFFT v7.310 [30], adding a total length of 10,698 bp. The best evolutionary model was obtained with JMODELTEST v2.1.10 [32], which used Akaike Information Criterion (AIC). The best model obtained was GTR + G, which was used to construct the phylogenetic tree based on ML in RAXML-HPC v8.2.10 [33] with 1000 replicates.

## 3. Results

After analyzing the complete chloroplast genomes of seven *Mammillaria* species, we estimated the different levels of molecular variation among the RSs, SSRs and divergent DNA sequences in homologous loci.

### 3.1. Repeated Sequences

**Figure 1:**
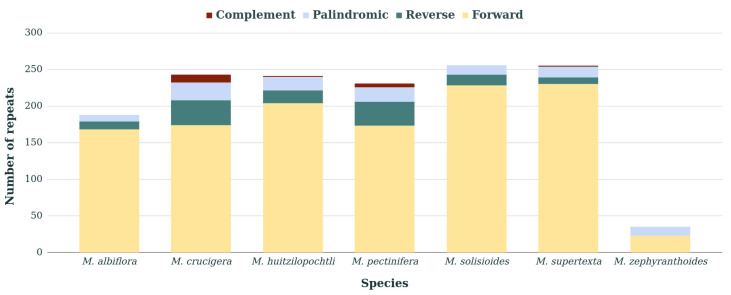
Number of repeated sequences (RSs) by type (complementary, palindromic, reverse and forward) identified in the seven species of *Mammillaria*.

Repeated sequences longer than 30 bp were found in the chloroplast genomes of the seven species of *Mammillaria*. The number of large RSs varied from 35 in *M. zephyranthoides* to 256 in *M. solisioides* (Figure 1). In *M. zephyranthoides,* we found only forward and palindromic RSs. In contrast, in the six other species, forward, reverse and palindromic RSs were detected. Additionally, *M. crucigera, M. pectinifera, M. huitzilopochtli* and *M. supertexta* had between one to 11 complement repeats. The forward RSs were the most abundant in the chloroplast genomes of the seven species (Figure 1). 

**Figure 2:**
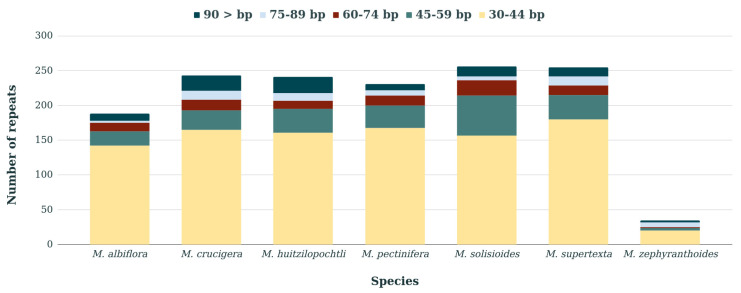
Number of repeated sequences identified in seven *Mammillaria* species. The color of the bars indicates the number of RSs by length in bp.

The number of RSs showed a tendency to decrease with the length of the repeated sequences in the seven species. The repeats from 30 to 44 bp represented >50% of the total repeated sequences in the seven species, whereas longer repeated sequences from 75 to 89 bp were the scarcest in *M. albiflora, M. crucigera, M. huitzilopochtli, M. pectinifera, M. solisioides* and *M. supertexta*. Likewise, in *M. zephyranthoides,* the less frequently repeated sequences were those from 60 to 74 bp (Figure 2).

### 3.2. Variation and Localization of the Short Sequence Repeats

We found a number of short sequence repeats ranging from 35 (*M. zephyranthoides*) to 84 (*M. pectinifera*) in the chloroplast genomes of the seven *Mammillaria* species, with different lengths and nucleotide compositions (Table 1 and Table S2). The SSRs that were composed of a single nucleotide (i.e., homopolymer repeats) or two nucleotides (i.e., dinucleotide repeats) were the most abundant, and they were detected in the seven species. On the other hand, the SSRs composed of three nucleotides were scarce; however, five species (*M. albiflora, M. crucigera, M. pectinifera, M. solisioides* and *M. supertexta*) contained one to four of any of the three types of trinucleotide repeat sequences (Table 1). Lastly, SSRs composed of four nucleotides (i.e., tetranucleotide) were identified only in *M. crucigera* and *M. supertexta*, whereas repeated sequences composed of five nucleotides were only found in *M. solisioides*, and those SSRs with six nucleotides were only present in *M. zephyranthoides.*

**Figure 3:**
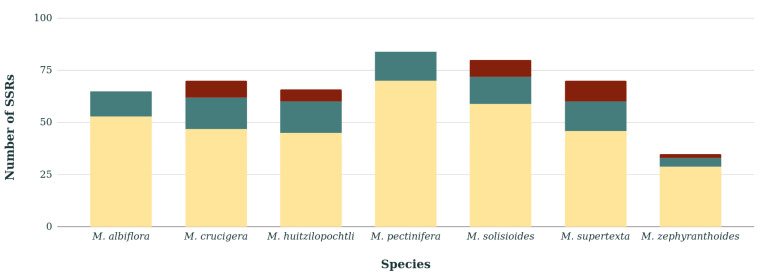
Total number of Short Sequence Repeats (SSRs) identified in the cpDNA of seven species of *Mammillaria*. The color of the bars corresponds to Inverted Repeats (red), Large Single Copy (yellow) and Small Single Copy (green).

Most of the SSRs were identified in the LSC region of the chloroplast genome of the seven species, whereas SSRs were scarce in the IRs, and particularly in *M. albiflora* and *M. pectinifera* they were absent in the IRs (Figure 3). Within the homopolymer repeats, we found five homologous SSRs that were shared in the seven species, and of these only three had length variations between species (Table S3).

### 3.3. Divergent DNA Sequences in Homologous Loci

**Figure 4:**
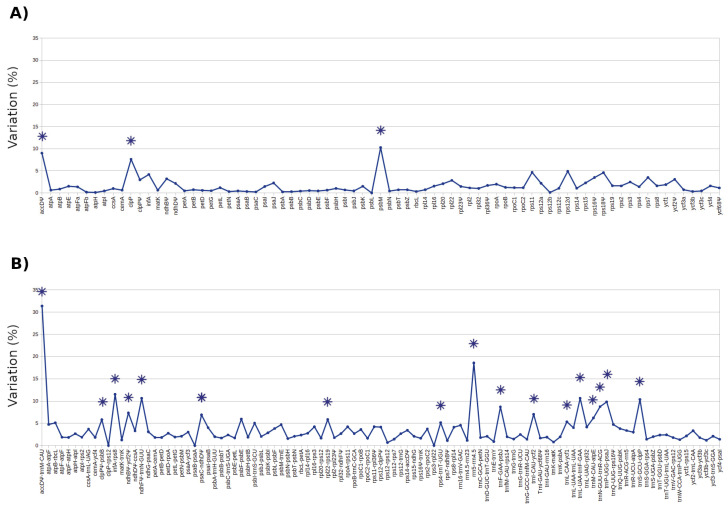
Distribution of the percentage of molecular variation across 180 homologous loci. Average percentage variation of the (**A**) coding and (**B**) non-coding homologous loci in the seven chloroplast genomes of *Mammillaria.* The 20 loci with the highest molecular variation are indicated with asterisks (✴).

In the seven species that were analyzed, a total of 180 homologous DNA loci were identified (Figure 4). Among them, 77 were coding and 103 were non-coding loci. The paired comparisons showed that coding loci had a molecular variation that oscillated from 0 to 10.26%, and non-coding loci showed levels from 0 to 31.37% (Figure 4A,B and Table S4).

Of the 180 homologous loci, ~77% were located in the LSC region of the chloroplast genome, ~16% in the SSC region and ~7% in the IRs. Since the seven *Mammillaria* species had three distinct cpDNA arrangements, the number of loci in each structural region differed between species (Table 2).

Among the 180 loci, we identified the 20 loci with the highest average levels of molecular variation (Figure 4), ranging from 5.14 to 31.37% (Table 3). Among these loci, 17 were IGS, and three were genes or potential pseudogenes.

## 4. Discussion

In this work, we evaluated the chloroplast genomes of seven *Mammillaria* species (*M. albiflora, M. crucigera, M. huitzilopochtli, M. pectinifera, M. solisioides, M. supertexta* and *M. zephyranthoides)*, in order to characterize their genetic variation and identify potential molecular markers for evolutionary biology studies. Our results showed that the high levels of quantified variation might enable the resolution of phylogenetic relationships between the species and genus levels. 

A previous study found that there are structural variations in the *Mammillaria* chloroplast genomes, identifying three different arrangements in the seven analyzed species [20]. Repeated DNA sequences have been proposed to produce chloroplast genome instability [34]. This idea is based on empirical evidence that plant species with strongly rearranged chloroplast genomes have a larger proportion of repeated sequences than those without a structural rearrangement [35,36]. However, the relatively low proportions of repeated sequences detected in *M. zephyranthoides* do not support such a notion, since a previous study documented that *M. zephyranthoides* shows deep and strong structural changes [20]. Our results do not allow for the conclusion that, in *Mammillaria,* the type of IRs is correlated to the number of RS contained in them. 

With respect to SSRs, these have been used for a long time as a source of molecular variation. In particular, they show high mutation rates at the intraspecific level [37], mainly due to variations in length [38]. In the cpDNA of *Mammillaria* here analyzed, homopolymer SSRs were the most abundant, and SSRs with >3 nucleotides were scarce or absent, as has been reported in other chloroplast genomes of angiosperms [39,40]. In addition, we concluded that the length and the number of genes involved in the formation of IRs are not the factors that determine the abundance of SSRs, since these were absent in IRs of *M. albiflora* and *M. pectinifera* that have only three genes and a length of <1000 bp [20]. However, *M. zephyranthoides* has divergent IRs of 14 kb and 21 or 22 genes [20], but only contains two SSRs (Figure 3).

In addition, we searched for the variation in SSRs among the seven species, in order to identify potential molecular markers. However, only five SSRs were identified in the seven species, and only three of them showed variation between species (Table S3). Thus, we do not recommend the use of SSRs of the chloroplast genome as unique molecular markers to resolve phylogenetic, phylogeographic or population issues, but these may be used together with other markers. These SSRs may be used not only for *Mammillaria* species, most of them being included in the IUCN Red List [25], but also extensively for the remaining Cactaceae and Caryophyllales members. 

The homologous loci are potential sources of variation that should be examined and selected according to the problem that is to be resolved (i.e., population or phylogenetic levels), since the levels of variation differ among them. In particular, there are 20 loci that showed a high molecular variation. We encourage their use for undertaking phylogenetic studies, as well as phylogeographic and population genetics studies. Notably, seven of these loci showed variation levels >10%, which represents an unusually higher level of molecular variation, and, consequently, they might be further tested in population level studies. Indeed, we consider that these loci may be tested in those angiosperms that show rapid radiations or a recent origin, since these are conditions that reduce molecular variation [41,42]. In the particular case of *Mammillaria*, its recent origin (<8 Ma) [26,27] could probably be causing the low levels of variation found in the few markers conventionally used in the preliminary phylogenetic studies carried out on both Cactaceae and *Mammillaria*. However, our results showed that, despite the recent origin of *Mammillaria* [26,27], its chloroplast genomes have DNA sequences with high levels of molecular variation, which were evident only by using the perspective of comparative genomics. Out of the 20 most variable loci here proposed as a source of molecular variations, only the *trnL-trnF* IGS, with an average 10.64% of divergence, was previously used, in a phylogenetic study that analyzed 21 species of *Mammillaria* [15]; however, this marker did not resolve relationships. Based on these results, we recommend a multiloci sampling to improve the resolution of the analysis and expand the taxonomic sampling. Moreover, 12 of the 20 most variable loci analyzed in this paper have amplicons (150–1000 bp) that can be sequenced via the Sanger method. Accordingly, these loci could be easily obtained at relatively low costs. In addition, out of convention, two IGS (*trnK-matK, psbA-trnH*) have been recommended and widely used for phylogenetic studies because they were considered as having a high molecular variation in angiosperms [43,44]. They have also been used in *Mammillaria* [23], with poor results. Consequently, we do not recommend the use of these two IGS as molecular markers, since they showed low percentages of variation (0.8 and 2%, respectively).

**Figure 5:**
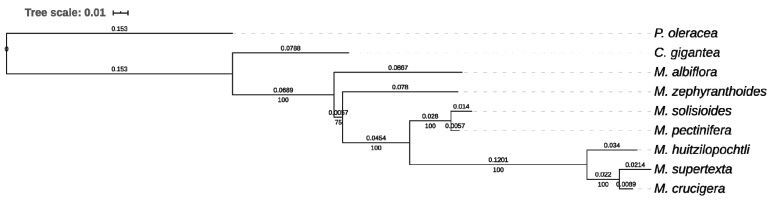
Phylogenetic ML tree for the seven studied *Mammillaria* species based on 14 loci of high molecular variation (marked with an asterisk in Table 3), with *Portulaca oleracea* and *Carnegiea gigantea* used as outgroups. The branch length is shown above the branches, whereas bootstrap values are displayed below the branches.

In order to test the feasibility of using the homologous loci values as molecular markers because of their high variation, we used 14 of them to construct a phylogenetic tree (Figure 5). The tree we obtained showed a topology similar to the one that had been previously obtained using 42 coding regions [20]. This result suggests that the small number of molecular markers here proposed could reduce the costs in phylogenetic studies with satisfactory results. Lastly, we encourage the testing of these 20 loci as molecular markers, in order to resolve the unclear limits of genera such as *Coryphantha, Escobaria, Neolloydia, Ortegocactus* and *Pelecyphora*. In addition, the use of these markers may also be considered for undertaking phylogenetic studies in other land flowering plants that require different options for molecular markers with high levels of variation.

## 5. Conclusions

In this study, the comparison of the complete chloroplast genome revealed new loci of high molecular variation in *Mammillaria* that were overlooked without a comparative genomics perspective. The high levels of molecular variation of these loci may open a new era in phylogenetic, population and taxonomic studies, as well as, in conservation issues with *Mammillaria* genus, the richest taxa of Cactaceae.

## Figures and Tables

**Table 1:** Type and number of Short Sequence Repeats (SSRs) in the complete chloroplast genomes of the seven analyzed species.

Type of SSRs	*M. albiflora*	*M. crucigera*	*M. huitzilopochtli*	*M. pectinifera*	*M. solisioides*	*M. supertexta*	*M. zephyranthoides*
1. Homopolymer							
A/T	56	60	57	69	65	60	31
C/G	1	1	1	2	2	1	0
2. Dinucleotide							
AT/AT	5	6	7	9	9	6	3
3. Trinucleotide							
AAG/CTT	2	1	0	3	2	1	0
AGG/CCT	1	0	0	0	0	0	0
AAT/ATT	0	0	0	1	1	0	0
4. Tetranucleotide							
AAAT/ATTT	0	2	0	0	0	2	0
5. Pentanucleotide							
AATAT/ATATT	0	0	0	0	1	0	0
6. Hexanucleotide							
ACGAGG/CCTCGT	0	0	0	0	0	0	1
Total number of SSRs							
	65	70	65	84	80	70	35

**Table 2:** Distribution of the 180 homologous loci identified in the seven *Mammillaria* species. The number of loci in each structural region of the chloroplast genome is shown.

Species	Number of Loci		
	LSC Region	SSC Region	IRs Region
Arrangement 1 (*M. albiflora* and *M. pectinifera*)	144	132	142
Arrangement 2 (*M. crucigera, M. huitzilopochtli, M. solisioides* and *M. supertexta*)	34	34	17
Arrangement 3 (*M. zephyranthoides*)	2	14	21

**Table 3:** Percentage of variation and length of the 20 most variable chloroplast loci recorded in the seven *Mammillaria* species. The type of locus (gene, pseudogene or IGS) and the structural region of the genome where it is located are shown. SSC/IRs indicates that the locus is in the SSC region in all the species, except *M. zephyranthoides*, which has it in IRs. * indicates the 14 loci that were used to obtain the phylogenetic relationships of the seven studied species. The last column represents the average length (bp) in the seven species.

Locus Name	Locus Type	Location	Variation Percentage (%)	Average Length (bp)
1. *accD-trnM* *	IGS	LSC	31.37	2176
2. *rrn5-rrn4.5* *	IGS	SSC	18.56	721
3. *infA-rps8* *	IGS	LSC	11.52	103
4. *trnL-trnF* *	IGS	LSC	10.64	325
5. *ndhF-trnN*	IGS	SSC	10.63	455
6. *trnS-clpP*	IGS	LSC	10.34	89
7. *psbM* *	Gene	LSC	10.26	102
8. *trnP-psaJ* *	IGS	LSC	9.82	399
9. *accDψ*	Pseudogene	LSC	8.99	1574
10. *trnN-trnR* *	IGS	SSC/IRs	8.74	360
11. *trnF-psbJ*	IGS	LSC	8.68	619
12. *clpP* *	Gene	LSC	7.61	616
13. *ndhB-ycf2*	IGS	SSC/IRs	7.34	354
14. *trnI-ycf2* *	IGS	SSC/IRs	7.02	109
15. *psaC-ndhD* *	IGS	SSC	6.90	146
16. *trnM-atpE* *	IGS	LSC	6.21	227
17. *rpl22-rps19* *	IGS	LSC	5.87	67
18. *clpP-psbB* *	IGS	LSC	5.82	753
19. *trnL-ycf1*	IGS	SSC	5.34	256
20. *rps4-trnT* *	IGS	LSC	5.14	277

## Supplementary Materials

The following are available online at https://www.mdpi.com/2073-4425/11/7/830/s1. Table S1: The species of *Mammillaria* analyzed in this study with their respective GeneBank accession number. Table S2: Nucleotide composition of SSRs (in parenthesis) and number of repeats (out of parenthesis) identified in the complete chloroplast genome of seven *Mammillaria* species. The location (start-end) of these SSRs is referred to the DNA sequences with the accession number shown in Table S1. Table S3: Composition and location of the five homologous SSRs identified in the seven *Mammillaria* species. Table S4: Percentage of molecular variation estimated between the seven *Mammillaria* species in paired comparisons of homologous loci (77 coding regions, followed by 103 non-coding regions).
