# Supplementary material for: Identification of High Molecular Variation Loci in Complete Chloroplast Genomes of Mammillaria (Cactaceae, Caryophyllales)
Source: Genes (Basel). 2020 Jul 21;11(7):830. doi: 10.3390/genes11070830 (PMC7397273; doi:10.3390/genes11070830)
Supplement: Supplementary file 1 [file genes-11-00830-s001.zip › Supplementary_files/Table_S1_Mammillaria_Chincoyaetal.docx]

Table S1. The species of *Mammillaria* analyzed in this study with their respective GeneBank accession number

| **Species** | **Accession Number** |
| --- | --- |
| *M. albiflora* | MN517610 |
| *M. crucigera* | MN517613 |
| *M. huitzilopochtli* | MN517612 |
| *M. pectinifera* | MN519716 |
| *M. solisioides* | MN518341 |
| *M. supertexta* | MN508963 |
| *M. zephyranthoides* | MN517611 |
