# Supplementary material for: Identification of High Molecular Variation Loci in Complete Chloroplast Genomes of Mammillaria (Cactaceae, Caryophyllales)
Source: Genes (Basel). 2020 Jul 21;11(7):830. doi: 10.3390/genes11070830 (PMC7397273; doi:10.3390/genes11070830)
Supplement: Supplementary file 1 [file genes-11-00830-s001.zip › Supplementary_files/Table_S3_Mammillaria_Chincoyaetal.docx]

Table S3. Composition and location of the five homologous SSRs identified in the seven *Mammillaria* species.

| Repeat motif | | | | | | | Location |
| --- | --- | --- | --- | --- | --- | --- | --- |
| *M. albiflora* | *M. crucigera* | *M. huitzilopochtli* | *M. pectinifera* | *M. solisioides* | *M. supertexta* | *M. zephyranthoides* |  |
| (T)_10_ | (T)_10_ | (T)_10_ | (T)_10_ | (T)_10_ | (T)_10_ | (T)_10_ | *rpoC2* |
| (A)_12_ | (A)_15_ | (A)_12_ | (A)_14_ | (A)_13_ | (A)_13_ | (A)_11_ | *trnC* |
| (A)_10_ | (A)_10_ | (A)_10_ | (A)_10_ | (A)_10_ | (A)_10_ | (A)_10_ | *psbF* |
| (T)_13_ | (T)_10_ | (T)_15_ | (T)_15_ | (T)_12_ | (T)_10_ | (T)_11_ | *petL-petG* |
| (T)_11_ | (T)_10_ | (T)_13_ | (T)_11_ | (T)_10_ | (T)_13_ | (T)_11_ | *rps11-rpl36* |
